# The Liquid Guide System: Design and Clinical Feasibility of an Internally Irrigated 3D-Printed Surgical Guide for Implant Surgery

**DOI:** 10.3390/jcm15145468

**Published:** 2026-07-13

**Authors:** Adrian Teodor Moga-Rogoz, Tudor Matei, Mihaela Hedeșiu, Mihaela Băciuț, Sorana Eftimie, Marius Steigmann

**Affiliations:** 1 Department of Radiology, Faculty of Dental Medicine, Iuliu Hațieganu University of Medicine and Pharmacy, 400006 Cluj-Napoca, Romania; tudor.matei@mrdentalmed.ro; 2 Department of Oral and Maxillofacial Surgery and Radiology, Iuliu Hațieganu University of Medicine and Pharmacy, 32 Clinicilor Street, 400006 Cluj-Napoca, Romania; mhedesiu@umfcluj.ro (M.H.); mbaciut@umfcluj.ro (M.B.); 3 Steigmann Institute, 69151 Neckargemund, Germany; drsteigmann@gmail.com

**Keywords:** dental implants, guided surgery, osteonecrosis, internal irrigation, dental implantology, digital

## Abstract

**Background/Objectives**: Thermal injury during implant osteotomy is a recognized complication of guided surgery. Closed guide architectures and metallic sleeves restrict coolant access to the drill–bone interface, reducing the effectiveness of conventional external irrigation. This study aimed to describe the design and fabrication of a 3D-printed surgical guide system with integrated internal irrigation channels and to evaluate its technical and clinical feasibility in guided implant surgery. **Methods**: The system was developed by integrating CBCT and IOS data into BlueSky Bio planning software. Irrigation channels were generated by CAD subtraction. Guides were fabricated by additive manufacturing—digital light processing (DLP) in biocompatible Asiga DentaGuide resin and, in one case, selective laser melting (SLM) in Mediloy metal—with metal auxiliary components incorporated where required. Channel patency was confirmed by flow equivalence testing and visual inspection. The design was evaluated for compatibility with the BioHorizons Pro Surgical Guide Kit and the Ticare Fidelis Kit. Clinical feasibility was assessed in two cases: maxillary All-on-6 rehabilitation and immediate implant placement in the esthetic zone. **Results**: Both guides were successfully fabricated with confirmed patent irrigation channels. Guide seating was verified through occlusal fenestrations and secured with fixation pins. Irrigation flow remained unobstructed throughout all osteotomy steps, and no guide-related or irrigation-related complications were observed. The system was integrated into standard drilling protocols without modification for either implant platform. **Conclusions**: This proof-of-concept study demonstrates the technical feasibility of an internally irrigated surgical guide across two implant systems and distinct clinical scenarios. The proposed design was successfully integrated into standard guided surgery workflows and maintained functional irrigation. Further in vitro thermal studies and prospective clinical investigations are required to evaluate its effect on temperature control.

## 1. Introduction

Thermal injury to bone during implant osteotomy remains a persistent concern in oral and maxillofacial surgery, particularly in guided implant procedures where guide structures can restrict irrigation access to the drill–bone interface. Excessive heat generation may lead to osteonecrosis, impaired osseointegration, and compromised primary implant stability. Intraosseous temperature rise is influenced by multiple factors, including drilling speed, axial force, bur geometry, cortical bone density, and the efficiency of coolant delivery. Therefore, maintaining effective irrigation is essential to prevent thermally induced tissue damage [1].

In conventional static-guided surgery, irrigation is delivered externally, yet guide sleeves frequently obstruct coolant flow to the osteotomy, reducing thermal control. Rivara et al. reported that limited coolant diffusion within closed guide designs can exacerbate heat accumulation, especially in flapless procedures, where mucosal thickness and sleeve height increase the distance between coolant entry and the bone surface [2] and Yung-Chuan Chen et al. evaluate bone temperature rise during the bone drilling process and assess the high-risk zone for potential thermal bone necrosis [3]. This has driven several engineering adaptations. Internal-irrigation drills deliver coolants through the bur, whereas modified guide architectures attempt to direct fluid beneath the sleeve barrier.

Delivering coolant internally, either through the drill body or from proximal outlets, has been reported to improve thermal control [4]. In controlled testing on porcine femoral cortical bone, Tuce et al. [5] demonstrated that guides incorporating an internal irrigation channel produced the lowest mean temperature rise during drilling, with all measurements remaining below the accepted thresholds for thermal injury. Their design also showed the narrowest temperature distribution, indicating more consistent thermal behavior than conventional cylindrical or open-sleeve guides.

Other studies, however, have found no significant differences between internal and external cooling under certain experimental conditions [6]. These conflicting results reflect variations in study design, including differences in bur sharpness, debris accumulation, and flushing protocols. Hang-Nga Mai et al. reported an increase in the heat generated during osteotomy owing to the reduced contact of cooling fluids with the drills [7].

Alternative guide geometries such as sleeveless or “windowed” designs have also been explored to improve coolant access when closed sleeves block irrigation [8]. Although these configurations can increase fluid contact with the cutting flutes, they may reduce mechanical stability and positional accuracy due to material deformation [1]. These findings highlight the influence of guide design on coolant delivery and thermal behavior during osteotomy [9]. Parvizi et al. showed that incorporating inlet and outlet channels into the guide structure enables continuous coolant circulation, improving debris evacuation and reducing heat accumulation [4]. Their in-vitro results showed that such channel-based systems outperform traditional external irrigation, particularly in dense bone where heat dissipation is most challenged.

Although several internally irrigated guide designs have been reported, most remain experimental and have been evaluated primarily under in vitro conditions. Clinical implementation within a standard digital workflow and compatibility with commercially available guided surgery systems remain insufficiently documented. Therefore, there is a need for designs that can integrate internal irrigation into routine guided implant surgery without requiring proprietary drilling systems or substantial workflow modifications. The aim of this study was to present the digital design workflow, additive manufacturing process, and preliminary clinical feasibility of the Liquid Guide System, a 3D-printed surgical guide integrating internal irrigation channels for direct coolant delivery to the drill–bone interface, evaluated across two implant systems and distinct clinical scenarios. Implant positioning accuracy was not an endpoint of this study.

The principal novelty of the proposed system lies in the integration of the irrigation pathway directly into the guide body during the CAD design phase. Unlike externally applied or retrofitted cooling solutions, the channel is conceived as an intrinsic architectural element of the guide, precisely positioned relative to the drill sleeve and fabricated as a single-unit structure within a standard digital workflow.

## 2. Methods

### 2.1. Digital Workflow and Guide Design

The design of the Liquid Guide System was based on standard digital workflows used for conventional static surgical guides. CBCT datasets were acquired using a Carestream CS 8200 3D system (Carestream Dental, Atlanta, GA, USA; 90 kV, 2.5 mA, 15 s exposure time, voxel size 150 µm). The working arch, antagonist arch, and occlusal relationship were recorded using additional silicone impressions. The resulting study models were digitized using the inEos X5 laboratory scanner (Dentsply Sirona, Bensheim, Germany). CBCT and surface scan data were imported into BlueSky Bio planning software (v4.13) and superimposed for virtual implant planning.

**Figure 1:**
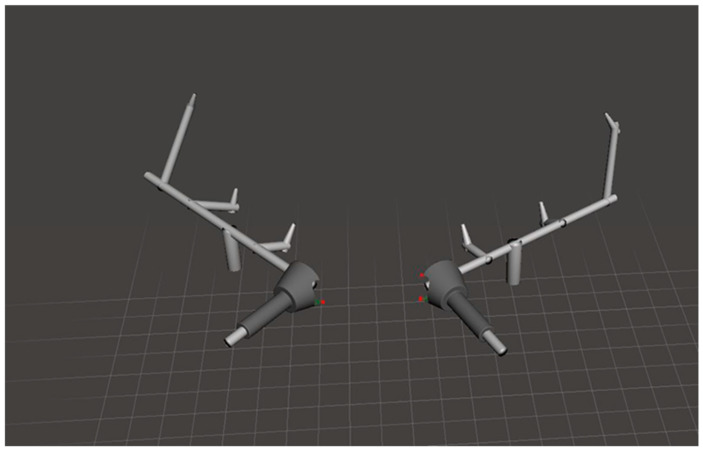
Digital irrigation channel concept—side-by-side parallel outlet prototype views.

**Figure 2:**
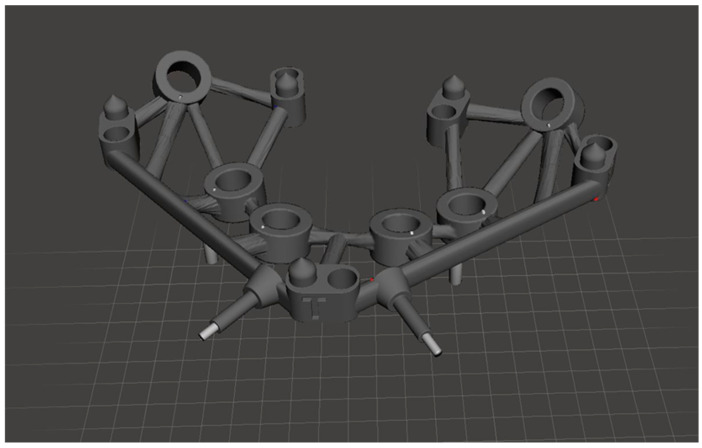
Complete CAD view of the finalized model used in full-arch guide fabrication.

**Figure 3:**
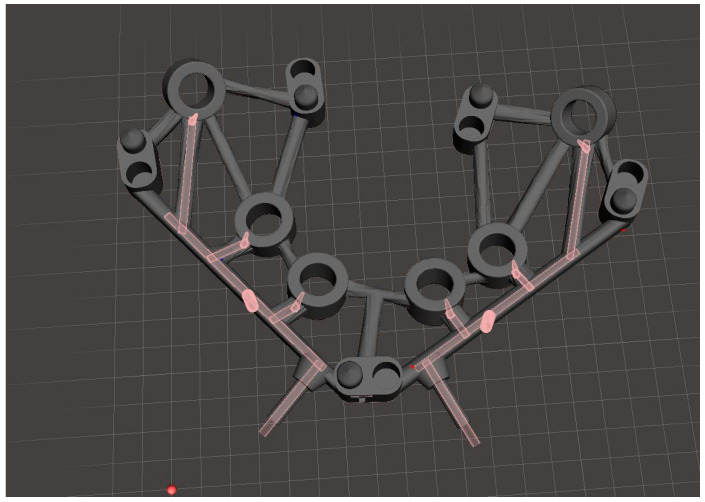
Highlighted internal microchannels visible within the model architecture.

Following implant planning, the surgical guide was designed within the same software. Internal irrigation channels were incorporated into the guide body using CAD subtraction, creating hollow pathways for saline delivery from the guide inlet to the osteotomy site (Figure 1, Figure 2, Figure 3 and Table 1).

Guide seating was verified through occlusal viewing fenestrations to confirm adaptation to the supporting dentition. Once positioning had been established, the guide was stabilized using metal fixation pins. The guide’s irrigation port was connected to the surgical micromotor’s saline source (physio dispenser) via a standard catheter, ensuring continuous flow through the guide. The drilling sequence adhered strictly to the protocol recommended by the implant system manufacturer.

The Liquid Guide was designed to integrate with two widely used guided-surgery platforms: the BioHorizons (Birmingham, AL, USA) implant system with its Pro Surgical Guide Kit and the Ticare (Valladolid, Spain) system with the Fidelis kit. Adaptation to the two systems required only minor modifications to sleeve geometry and guide dimensions during the CAD design process, while the irrigation channel remained unchanged.

### 2.2. 3D Printing and Material Selection

Guides were fabricated using digital light processing (DLP) technology with an Asiga MAX UV printer (Asiga, Sydney, Australia) and biocompatible Asiga DentaGuide photopolymer resin (Asiga, Sydney, Australia). The material is intended for surgical guide fabrication and maintains dimensional stability following steam sterilization. Based on previously reported fabrication thresholds [4], the internal irrigation channels were designed with a diameter of 0.8 mm to preserve channel patency while maintaining structural integrity. Auxiliary metal components were fabricated separately using an SLM/LMF MYSINT 100 3D printer (Sisma S.p.A., Piovene Rocchette, Italy) with Mediloy metal powder (BEGO Implant Systems, Bremen, Germany). In the post-processing phase, the guide was manually finished using abrasive instruments to remove printing excess.

In Case 1, the guide was fabricated using metal 3D printing (SLM/LMF MYSINT 100, Mediloy powder). In Case 2, the guide was fabricated from biocompatible DLP-printed resin (Asiga DentaGuide).

### 2.3. Irrigation Integration and Sterilization

**Figure 4:**
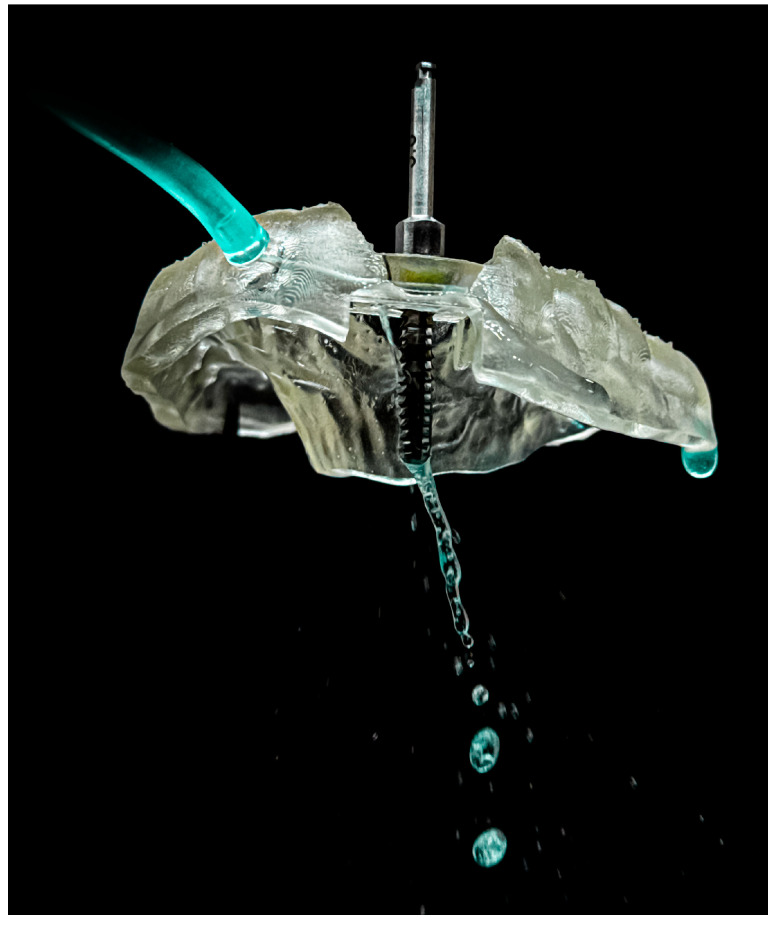
Illustration of saline flow through the resin Liquid Guide irrigation channel, shown for demonstrative purposes. Direction of the irrigation flow through the guide onto the cutting interface of the drill.

After printing, guides underwent steam autoclave sterilization according to standard protocols. Internal irrigation channels were visually inspected before and after sterilization to verify patency and the absence of obstruction or deformation. To verify that the integrated channel did not restrict saline delivery, irrigation flow was assessed by measuring the volume of saline dispensed over a fixed time interval (the measured volume in mL, the timed interval in s, and the number of repetitions, e.g., *n* = 3) under two conditions: free micromotor hose delivery and delivery through the connected Liquid Guide. The measured volume was 500 mL, the timed interval in 600 s, and the number of repetitions, e.g., *n* = 3, for free micromotor hose delivery and delivery through the connected Liquid Guide every time. The recorded volumes were equivalent, confirming that the guide does not impede coolant flow relative to standard irrigation. The guide inlet was connected directly to the saline delivery system of the surgical micromotor via standard tubing, enabling continuous irrigation through the integrated channel network (Figure 4). Irrigation was performed according to the manufacturer’s recommended drilling protocol for each implant system. Irrigation was delivered at a temperature of 7 °C of the saline solution using the surgical micromotor set to pump level 3 (medium flow). Flow equivalence testing confirmed that saline volume delivered through the integrated guide channel over a fixed time interval was identical to that of the free micromotor hose, simultaneously validating both channel patency and adequate flow rate.

### 2.4. Clinical Application and Workflow Integration

To evaluate the technical and clinical feasibility of the proposed design, the Liquid Guide System was applied in two surgical cases representing distinct clinical scenarios: Case 1 (Figure 1, Figure 2, Figure 3 and Figure 5, Figure 6, Figure 7, Figure 8) and Case 2 (Figure 4 and Figure 9, Figure 10, Figure 11). Both cases were planned and executed following the same digital workflow described above.

## 3. Results

Both surgical guides were successfully fabricated with patent internal irrigation channels. Patency was confirmed by visual inspection after printing and following autoclave sterilization and was further validated by unobstructed irrigation flow during clinical use. No lumen collapse, resin debris, or dimensional deformation was observed in either guide. The minimum channel diameter of 0.8 mm was maintained throughout both designs, consistent with the fabrication tolerances of the additive manufacturing processes used (DLP and SLM).

### 3.1. Case 1: Maxillary “All-on-6” Implant-Prosthetic Rehabilitation

A 54-year-old female patient presented for complex oral rehabilitation. Her general medical history revealed the presence of hepatitis and hypercholesterolemia, managed with specific medication (Sortis, Lipantil), alongside a known allergy to Chloramphenicol. Clinical and radiographic diagnosis highlighted a maxillary Kennedy Class II subclass 1 edentulism (partially restored anteriorly) and generalized periodontitis, Stage III, Grade B. The treatment plan involved the extraction of the compromised remaining teeth and fixed implant-prosthetic rehabilitation of the maxilla utilizing the “All-on-6” concept. Employing the Liquid Guide system for optimized thermal control during osteotomy, six Ticare^®^ Inhex Quattro implants (Mozo-Grau S.A., Valladolid, Spain) were placed using a fully guided approach. Implant dimensions were adapted to the local bone availability as follows: in positions 1.6 (3.75/15 mm), 1.4 (3.75/13 mm), 1.2 (3.75/13 mm), 2.1 (3.75/13 mm), 2.3 (3.75/15 mm), and 2.6 (3.75/15 mm).

**Figure 7:**
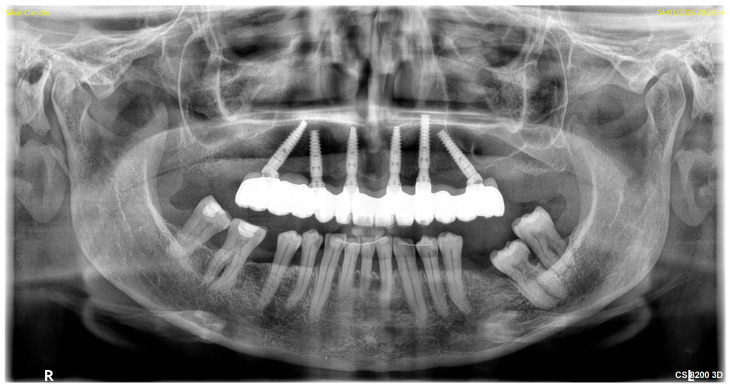
One-year follow-up panoramic radiograph (Case 1).

Guide seating was confirmed through occlusal fenestrations and secured with fixation pins. Saline irrigation flow was unobstructed throughout all six osteotomies. No intraoperative complications related to the guide or irrigation system were recorded. The 1-year follow-up panoramic radiograph demonstrates stable clinical results, with no radiographic evidence of pathological peri-implant bone loss (Figure 7).

**Figure 8:**
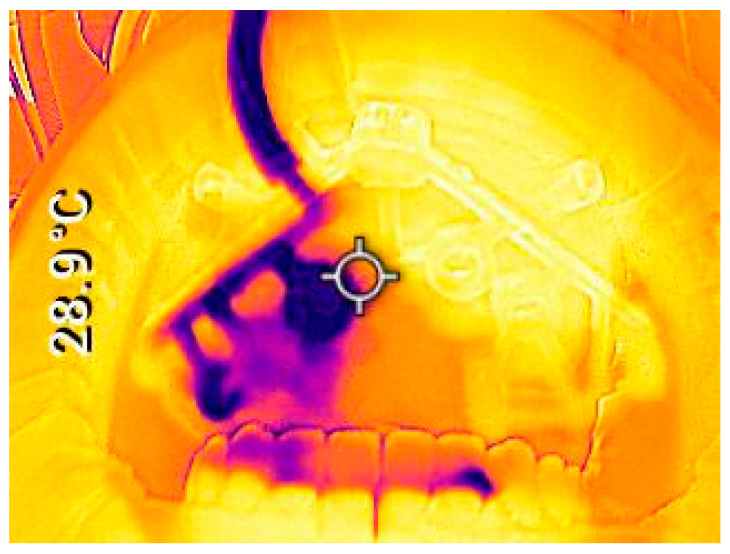
FLIR thermal imaging during Case 1 osteotomy, showing temperature distribution at the drill–bone interface and along the irrigation channels.

Intraoperative thermal imaging was performed with a FLIR camera to qualitatively monitor the drill–bone interface during osteotomy. The recorded thermal patterns were consistent with temperatures remaining within commonly accepted biological limits; however, absolute temperatures were not quantified in this proof-of-concept study (Figure 8).

### 3.2. Case 2: Extraction and Immediate Implant Placement in the Esthetic Zone

A 36-year-old, clinically healthy male patient presented complaining of mobility in the right maxillary central incisor (tooth 1.1). Clinical examination revealed a maxillary Kennedy Class III subclass 4 edentulism (restored), and grade 2 mobility was recorded at tooth 1.1 (previously endodontically treated). CBCT radiographic evaluation confirmed the complete resorption of the buccal cortical bone plate along the entire root length of tooth 1.1. The treatment plan involved the atraumatic extraction of the affected tooth and immediate dental implant placement. The intervention was performed using a Liquid Guide series surgical guide, placing a Tapered Internal Laser-Lok implant (BioHorizons, Birmingham, AL, USA) measuring 3.8/15 mm.

**Figure 10:**
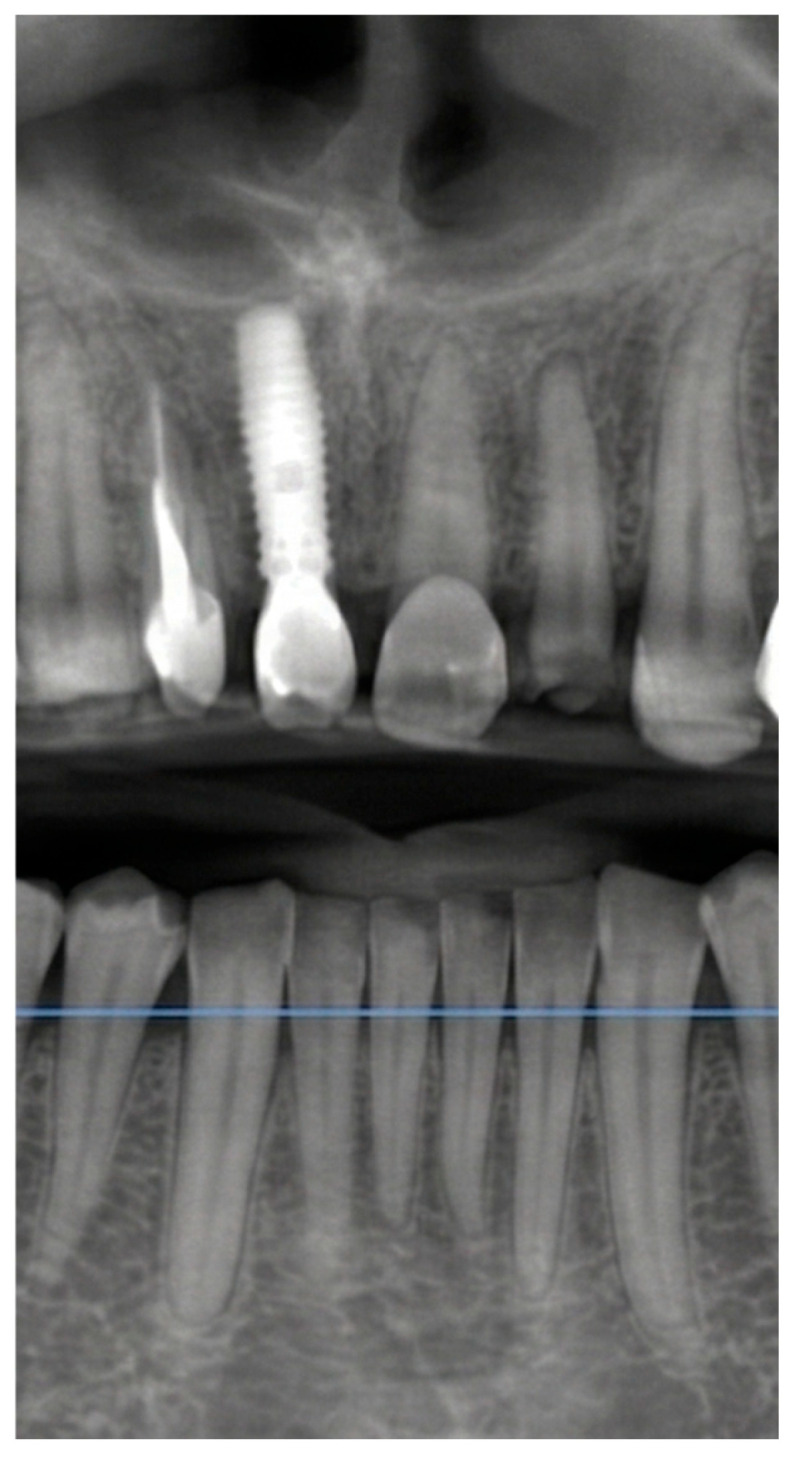
One-year follow-up panoramic radiograph (Case 2).

Similar favorable outcomes were observed in the second clinical case. At the 12-month post-operative mark, clinical and radiographic evaluations confirmed excellent stability of the fixtures. The 1-year follow-up panoramic radiograph showed stable crestal bone levels. No pathological marginal bone resorption or peri-implant radiolucency was observed at 1-year follow-up panoramic radiograph (Figure 10), consistent with successful osseointegration.

**Figure 11:**
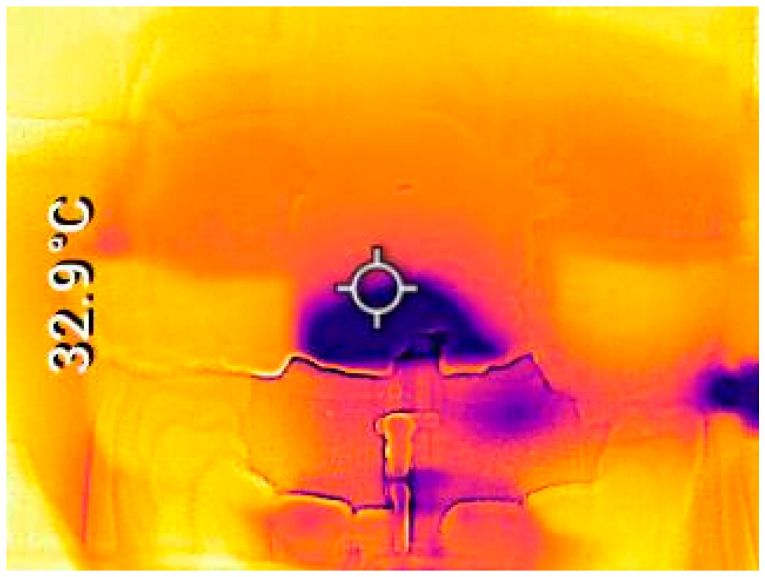
FLIR thermal imaging during Case 2 osteotomy, showing temperature distribution at the drill–bone interface and along the irrigation channels.

Thermal imaging in Case 2 similarly showed patterns consistent with controlled temperatures at the osteotomy site, without visual evidence of heat accumulation beyond accepted thresholds; absolute temperatures were not quantified (Figure 11).

## 4. Discussion

This study presents the design, digital fabrication workflow, and pilot clinical integration of a 3D-printed surgical guide incorporating internal irrigation channels for direct coolant delivery during implant osteotomy. The system was implemented in two clinical cases, both of which demonstrated favorable clinical and radiographic outcomes during follow-up.

Effective irrigation during implant osteotomy is essential to prevent friction-induced temperature rise above 47 °C. Beyond this threshold, collagen integrity, vascularization, and osteoblast viability become compromised [3,10]. In guided surgery, metallic sleeves partially enclose the drill–bone interface and can obstruct coolant flow, making external irrigation alone insufficient to maintain safe thermal conditions.

Overheating could cause necrosis, fibrosis, caustic bone degeneration, and usually decreased osteoblastic activity (Scarano et al., 2020) and the increased temperature impact caused by the adoption of the guiding procedure can be detrimental [11,12].

Early static surgical guides improved drill trajectory accuracy but often restricted irrigation because of their sleeve design. To address this limitation, sleeveless and partially constrained guides were introduced to improve coolant access. However, these designs may compromise guide stability and have shown inconsistent thermal performance. More recently, advances in additive manufacturing have enabled high-resolution 3D-printed guides with embedded internal channels [7]. These channels can direct coolant to regions that are difficult to reach with conventional external irrigation.

Internal or targeted irrigation systems have been proposed to improve coolant delivery by directing fluid toward the cutting flutes [13]. Studies by Haider and Plenk [14] highlight that compact cortical bone exhibits low tolerance to surface heating, making uniform coolant contact critical. They also reported better performance with combined internal and external cooling systems compared with either method alone. However, suboptimal channel design may increase the risk of outlet blockage by debris.

The Liquid Guide System builds on these developments by integrating internal irrigation channels into a standard digital workflow for guided implant surgery. The design is compatible with commercially available guided surgery systems and does not require proprietary drilling instruments.

To evaluate this approach, the system was implemented using two established implant systems: the BioHorizons implant system with its Pro Surgical Guide Kit and the Ticare implant system with the Fidelis guided surgery kit. These platforms were selected for their widespread clinical use and compatibility with 3D-printed guides. Adapting the design to the two systems requires only minor modifications to sleeve geometry and guide dimensions during the CAD design process. The internal channel configuration remained unchanged.

The clinical workflow begins with CBCT imaging and intraoral scanning, which together capture the hard- and soft-tissue anatomy required for prosthetically driven implant planning. Integration of surface texture data from intraoral scans may further improve the accuracy of guide seating [15]. Following virtual planning, the guide is fabricated using biocompatible resin that can withstand autoclave sterilization without deformation [4]. Nevertheless, preoperative verification of channel patency remains advisable [5].

Effective cooling is particularly important near the osteotomy margins, where heat generation is greatest and external irrigation may be limited [5]. The proposed design facilitates coolant delivery to these regions while maintaining the accuracy of guided surgery. Such considerations may be particularly relevant in anatomically demanding situations, including the esthetic zone [14].

Precise alignment between the irrigation channel outlets and the drill flutes is an important design consideration. Misalignment reduces irrigation efficiency by altering the direction of fluid flow toward the osteotomy site. Consequently, verification of guide seating and positional conformity remains an important step during clinical use [9].

### 4.1. Practical Considerations

The integrated irrigation system does not obstruct the surgical field or require manual coolant application. This may simplify the surgical workflow and reduce operative interruptions. Saline was delivered at 7 °C, which falls within the range recommended for maximizing cooling efficacy.

The proposed design is not restricted to a specific implant position or anatomical region. In the present study, the system was successfully applied in two distinct clinical scenarios involving different implant positions. Although broader validation is required, the design could be incorporated into standard guided surgery workflows across a range of clinical indications.

### 4.2. Future Directions

The following directions are proposed as future perspectives beyond the scope of the present study. Future studies should focus on quantifying the thermal performance of the proposed design through controlled in vitro experiments and larger prospective clinical investigations. Additional work is also needed to characterize irrigation flow, coolant distribution, and channel performance under different surgical conditions. Future iterations of the Liquid Guide System could incorporate temperature-monitoring sensors to provide real-time intraoperative feedback.

Several practical considerations remain important for clinical translation, including sterilization procedures, additive-manufacturing tolerances, and compatibility with different surgical platforms. Photopolymer resins must maintain dimensional stability and channel patency after sterilization, as repeated autoclave or plasma cycles may alter material properties [16]. Consistency in CAD-to-print manufacturing is equally important, since small deviations may affect guide fit and coolant flow. Incorporating patient identifiers onto guides for traceability could support compliance with medical device standards and facilitate clinical adoption [13].

### 4.3. Limitations

Several limitations should be acknowledged. Although intraoperative thermal measurements were obtained via FLIR imaging, formal quantitative analysis and larger prospective studies remain necessary to fully characterize the thermal performance of the system. Consequently, the effect of the proposed design on osteotomy temperature could not be quantified. Favorable clinical and radiographic outcomes were observed during follow-up.

Second, validation of the irrigation system was limited. Channel patency was confirmed by visual inspection before and after sterilization and by successful irrigation flow during surgery. However, additional validation methods, such as micro-CT analysis, dye penetration testing, pressure measurements, or quantitative flow assessment, were beyond the scope of this proof-of-concept study. Basic flow equivalence was confirmed; however, outlet pressure distribution and coolant dispersion at the drill–bone interface were not formally quantified.

The present findings should therefore be interpreted as evidence of technical feasibility and workflow integration. Future studies should incorporate quantitative thermal analyses, comprehensive validation of channel performance, and to further evaluate the clinical performance of the system. Such investigations will be necessary to establish the thermal performance, reproducibility, and potential clinical value of the proposed system.

## 5. Conclusions

Maintaining adequate cooling during guided implant osteotomy remains challenging, particularly when conventional sleeve designs limit irrigation access. The proposed 3D-printed guide incorporates internal irrigation channels intended to improve coolant access to the osteotomy site during drilling. Integration of CAD-based planning with standard intraoperative verification steps shows workflow compatibility and initial clinical feasibility. While the preliminary findings are encouraging, this proof-of-concept study was limited to a small number of clinical cases. Preliminary thermal imaging was obtained but requires further quantitative validation. Further in vitro and clinical studies are needed to evaluate the effect of this design on temperature control and to assess its performance across varied clinical scenarios, patient groups, and implant systems.

## Figures and Tables

**Figure 5:**
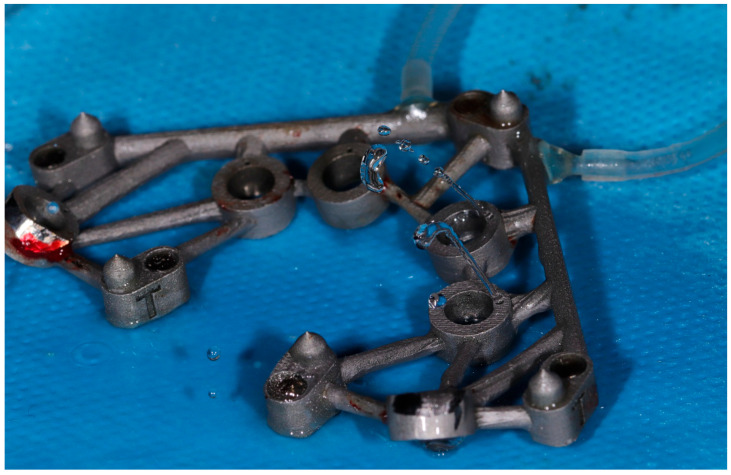
Metal 3D-printed guide with connected irrigation tubing during clinical placement (Case 1—”All-on-6” Implant—Prosthetic Rehabilitation).

**Figure 6:**
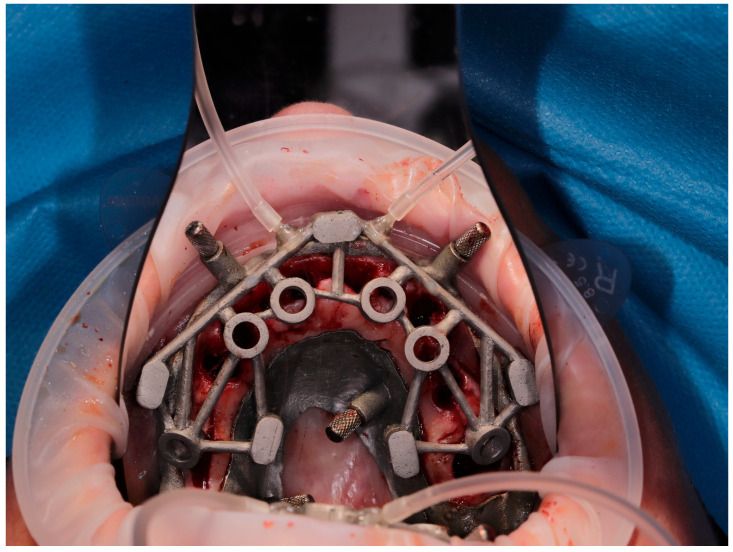
Functional irrigation shown on a printed guide with water flow visible (Case 1—”All-on-6” Implant—Prosthetic Rehabilitation).

**Figure 9:**
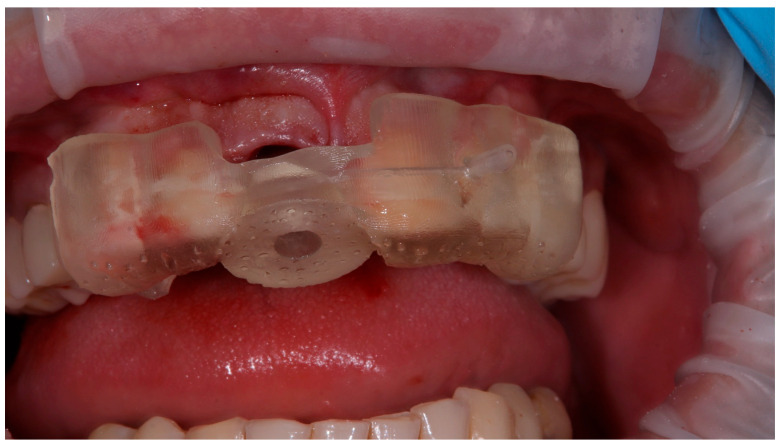
Surgical guide in clinical use showing the internal irrigation channel in place (Case 2—Immediate loading).

**Table 1:** Irrigation channel geometry of the Liquid Guide System.

Internal Channel Diameter	Inlet/Outlet Port Diameter	Channel Pathway Length	Channel Wall Thickness	Outlet Position Relative to Sleeve
0.8 mm	0.8 mm/0.2 mm	31 mm	0.9 mm	directed apically toward drill–bone interface

## Data Availability

The data presented in this study are not publicly available due to privacy and ethical restrictions (protecting the identity/confidentiality of the participants).
